# Allergic diseases of the skin and drug allergies – 2012. Is there association between duration of seasonal alleric rhinitis and oral allergy syndrome?

**DOI:** 10.1186/1939-4551-6-S1-P99

**Published:** 2013-04-23

**Authors:** Reza Faridhosseini, Farahzad Jabbari, Afshin Shirkani, Mohammad Reza Zandkarimi, Hadis Yousefzadeh

**Affiliations:** 1 Mashhad University of Medical Science, Mashhad, Iran

## Background

Oral allergy syndrome (OAS) characterized by oral IgE-Mediated symptoms, which is caused by cross-reactivity between proteins in pollen and fresh fruit or vegetables .Association between oral allergy syndrome and duration of seasonal allergic rhinitis, is not well known.

## Methods

In this cross sectional study, 103 consecutive patients with seasonal allergic rhinitis were enrolled. Sensitization to common aeroallergen confirmed by skin prick test 3 millimeter more than negative control. Clinical symptoms, personal and familial history of atopy and demographic data were listed. According to food allergy history 63 of 103 patients had positive Open Single-Blind Oral Food Challenge Test that was associated with oral allergy syndrome.

## Results

Among studied cases, 63 patients (61.2%) with 28.8±10.6 years old had OAS and 40 (38.8%) with 26.8±13.2 years had not OAS. We found that duration of seasonal allergic rhinitis in OAS group (7±5.9 years old) and non OAS group (5±4 years ) was significantly correlated (P=0.03).

## Conclusions

In this study, duration of seasonal allergic rhinitis was associated significantly with oral allergy syndrome. However further studies with more sample sizes and double blind placebo methods might be need.

